# Comment to: “One nose one brain: contribution of the main and accessory olfactory system to chemosensation” by Carla Mucignat, Marco Redaelli, Antonio Caretta

**DOI:** 10.3389/fnana.2012.00049

**Published:** 2012-12-11

**Authors:** Jorge A. Larriva-Sahd

**Affiliations:** Developmental Neurobiology, Neuromorphology, Universidad Nacional Autónoma de MéxicoQuerétaro, Mexico

**A commentary on**

**One nose one brain: contribution of the main and accessory olfactory system to chemosensation**

by Mucignat, C., Redaelli, M., and Caretta, A. (2012). Front. Neuroanat. **6**:46. doi: 10.3389/fnana.2012.00046

Current neurobiological evidence supports the synergic involvement of sensory organs in decoding complex environmental objects. The somewhat independent study of the anatomical and functional aspects of the major sensory systems has yielded a wealth of information on how environmental stimuli are decoded. Understanding the central integration that results in complex, behaviorally relevant objects, however, is still in its infancy. The same applies to chemical senses that act in concert to build representations of physiologically distinct objects which, in turn, motivate effective adaptive responses. The success of this chain of events is decisive for virtually all fundamental behaviors that lead to survival such as feeding, mating, fighting, or fleeing.

**Figure 1:**
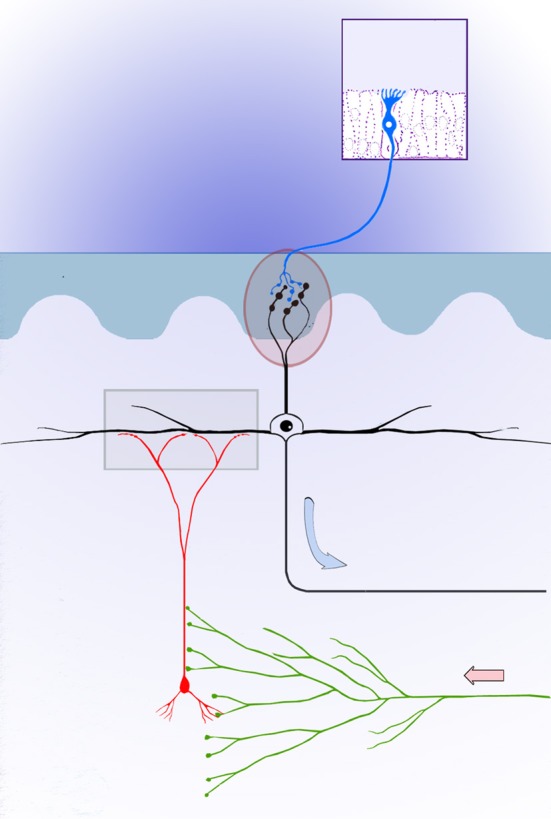
**Basic circuitry in the mammalian olfactory bulb.** Bipolar sensory receptor (blue) in the nasal cavity (upper box) whose axon interacts in the olfactory glomerulus (encircled) with the apical dendrite of a mitral cell (black); the axon (blue arrow) of the latter proceeds to the primary olfactory cortex. Centrifugal fibers (green) controlling a granule cell (red) modulate the activity of the mitral cell. It is currently assumed that dendro-denritic synapses between granule and mitral cell (lower box), provide the substratum for a reciprocal chemical modulation. Modified from Ramón y Cajal (1904).

A first approximation toward a holistic view of interrelated sensory senses is achieved by analyzing the molecular, developmental, and structural characteristics that underlie the function of each subsystem. In this broad context, the work by Mucignat-Caretta and coworkers describes the distinct, yet synergic, functions of the vomeronasal system and contrast them with other aspects of chemoreception associated with the face and its air-passages. This approach builds upon and challenges the early postulate regarding the functional dichotomy between the main and accessory olfactory systems, although the influence of endogenous axons (i.e., centrifugal fibers) (Figure 1) discovered by Ramón y Cajal (1904) provided a substratum for a central modulation of primary sensory organs and their relay areas and nuclei. Thus, the neural interaction between endogenous fibers with primary sensory targets represents the structural signature for a polymodal synergy.

Mucignat-Caretta and collaborators discuss intra-species recognition in the context of the ontogenetic onset and development of both pheromone and volatile substance decoding and the central thesis of this comprehensive revision. That is, the overlapping of volatile odorant and pheromonal responses. Emphasis is given to the detection of chemical cues by classic and new (e.g., Gruenberg ganglion) putative chemoreceptors.

In précis, the present article by Mucignat-Caretta et al. offers a concise account highlighting the most influential work supporting the integrative processes that take place between the chemical senses associated with volatile and pheromonal perception.
